# Myxoinflammatory fibroblastic sarcoma of the liver: Case report and literature review

**DOI:** 10.1097/MD.0000000000038796

**Published:** 2024-07-05

**Authors:** Yize Li, Luyao Zhang, Guona Zheng, Jing Li, Zhourun Ma, Xiuchuan Jia, Yingmin Chen

**Affiliations:** a Department of Radiology, Hebei General Hospital, Shijiazhuang, Hebei, China; b Department of Pathological, Hebei General Hospital, Shijiazhuang, Hebei, China; c Department of Graduate School, Hebei North University, Zhangjiakou, Hebei, China; d Department of Graduate School, Hebei Medical University, Shijiazhuang, Hebei, China.

**Keywords:** liver tumor, myxoinflammatory fibroblastic sarcoma, pathology, surgery

## Abstract

**Rationale:**

Myxoinflammatory fibroblastic sarcoma (MIFS) is a rare low-grade malignant soft tissue sarcoma that primarily affects the distal extremities in adults, with the highest incidence in patients in their 40s and 50s. It has a high local recurrence rate and a low metastasis rate. Although MIFSs have been documented in other sites, an MIFS in the liver is highly unusual. Herein, we present a case of a patient with hepatic MIFS.

**Patient concerns:**

The patient was a 58-year-old Chinese man with abdominal pain as the primary symptom. Abdominal computed tomography and magnetic resonance imaging revealed a mass in the right posterior lobe of the liver. The patient underwent surgical excision, and the excised specimen was identified as MIFS. Three years later, the patient returned to our hospital for abdominal pain. Computed tomography and magnetic resonance imaging revealed a mass in liver segments 2/3/4.

**Diagnosis:**

Postoperative pathological examination of the tumor revealed the recurrence of MIFS.

**Interventions:**

The patient underwent surgical resection of the MIFS.

**Outcomes:**

The patient received multiple pirarubicin-based chemotherapy treatments and an ALK inhibitor (anlotinib) within 6 months after surgery, but the tumor recurred.

**Lessons:**

MIFS can not only occur in the proximal limbs, trunk, head, and neck but can also affect the abdominal organs. Surgical resection remains the primary treatment option for MIFS in the absence of any contraindications. Because the recurrence rate of MIFS is high, meticulous long-term monitoring is required.

## 1. Introduction

Acral myxoinflammatory fibroblastic sarcoma was independently reported in 1998 by Michal,^[1]^ Montgomery,^[2]^ Meis-Kindblom, and Kindblom.^[3]^ It is a sporadic soft tissue tumor that primarily affects adults, with the highest incidence in their 40s and 50s, and the incidence is nearly equal between men and women. It often manifests as a painless mass at the distal end of the limb, primarily on the hands and feet, accounting for almost two-thirds of all cases.^[4]^ However, it can occur in multiple body parts, including the iris,^[5]^ parotid gland,^[6]^ maxilla,^[7]^ back,^[8]^ eyeball,^[9]^ nasal dorsum,^[10]^ scalp,^[11]^ and groin.^[12]^ As a result, the World Health Organization (WHO) renamed it myxoinflammatory fibroblastic sarcoma (MIFS) in 2002. In this study, we present a case of hepatic MIFS in a 58-year-old male. To the best of our knowledge, this is the first case of hepatic MIFS.

## 2. Case report

**Figure 1.**
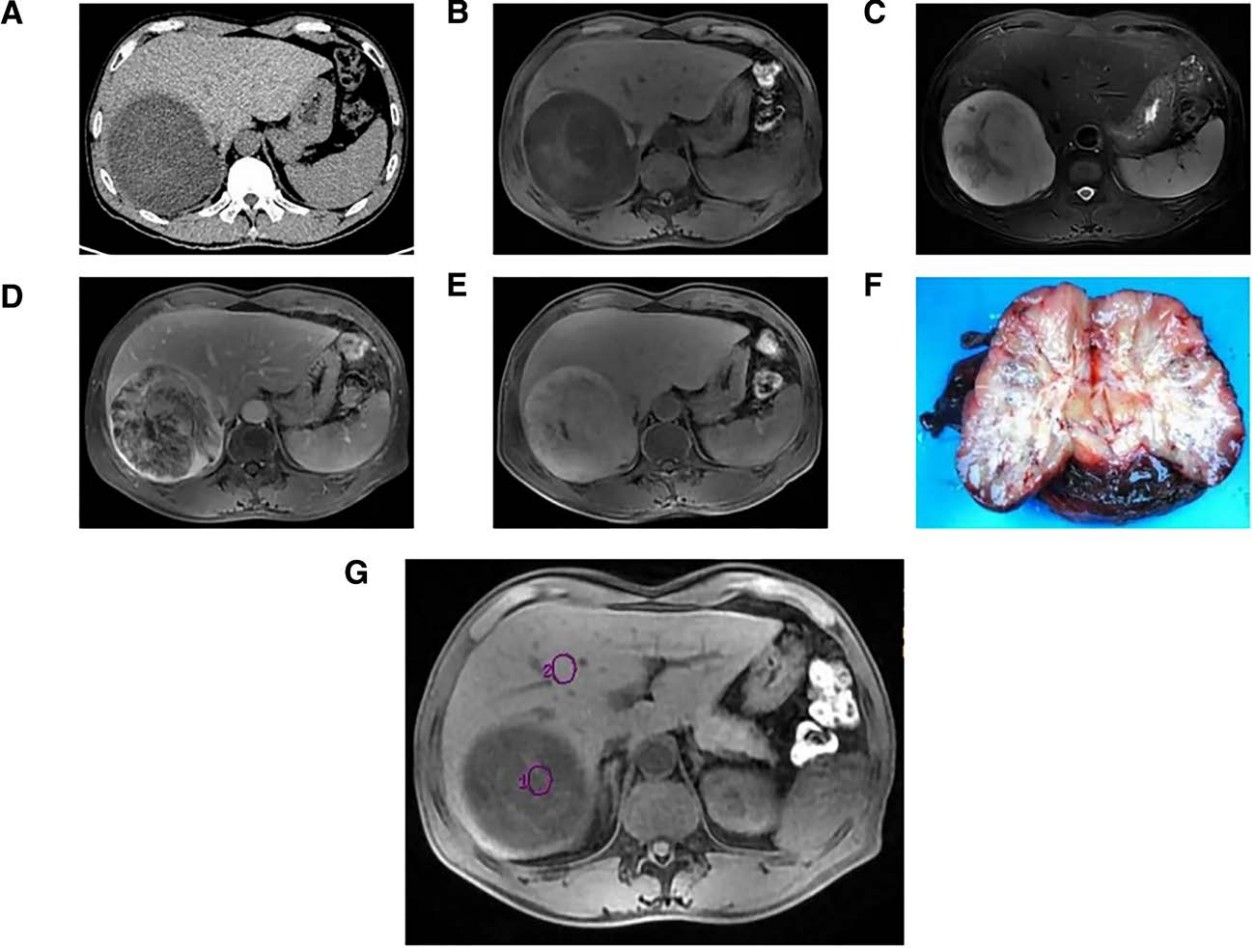
CT, MRI, and specimen of the tumor. (A) CT image. (B) T1-weighted image. (C) T2-weighted image. (D) Delayed phase. (E) Hepatobiliary phase. (F) Specimen. (G) The ROI of time–signal intensity curve of the tumor. CT = computed tomography.

**Figure 2.**
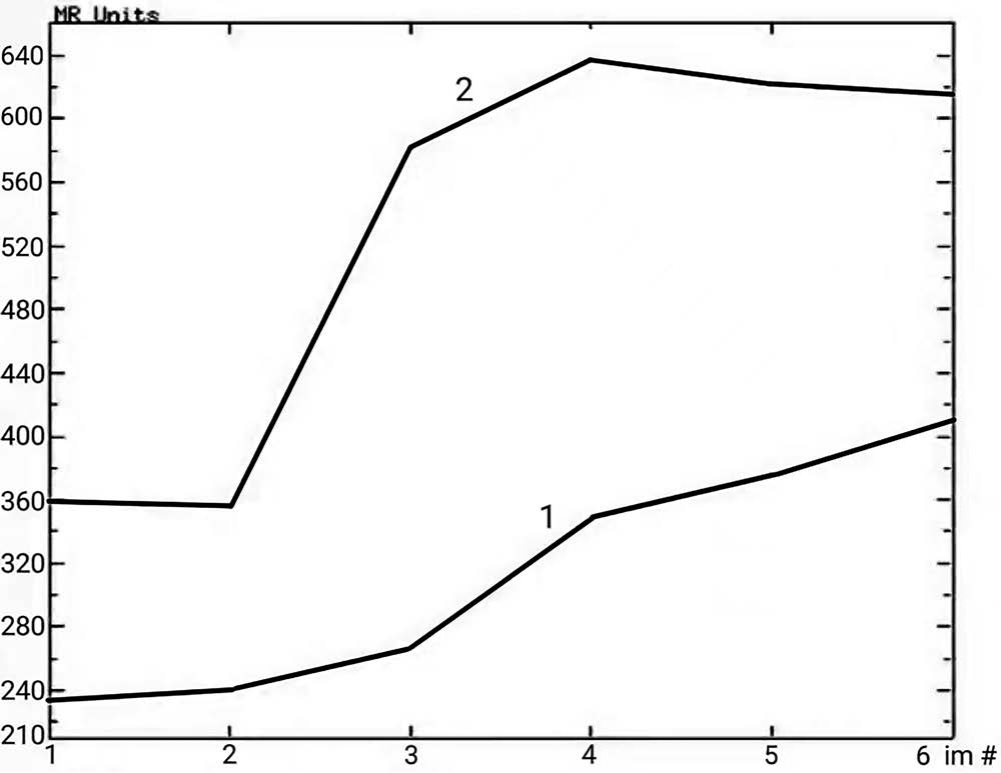
The time–signal intensity curve of the tumor.

**Figure 3.**
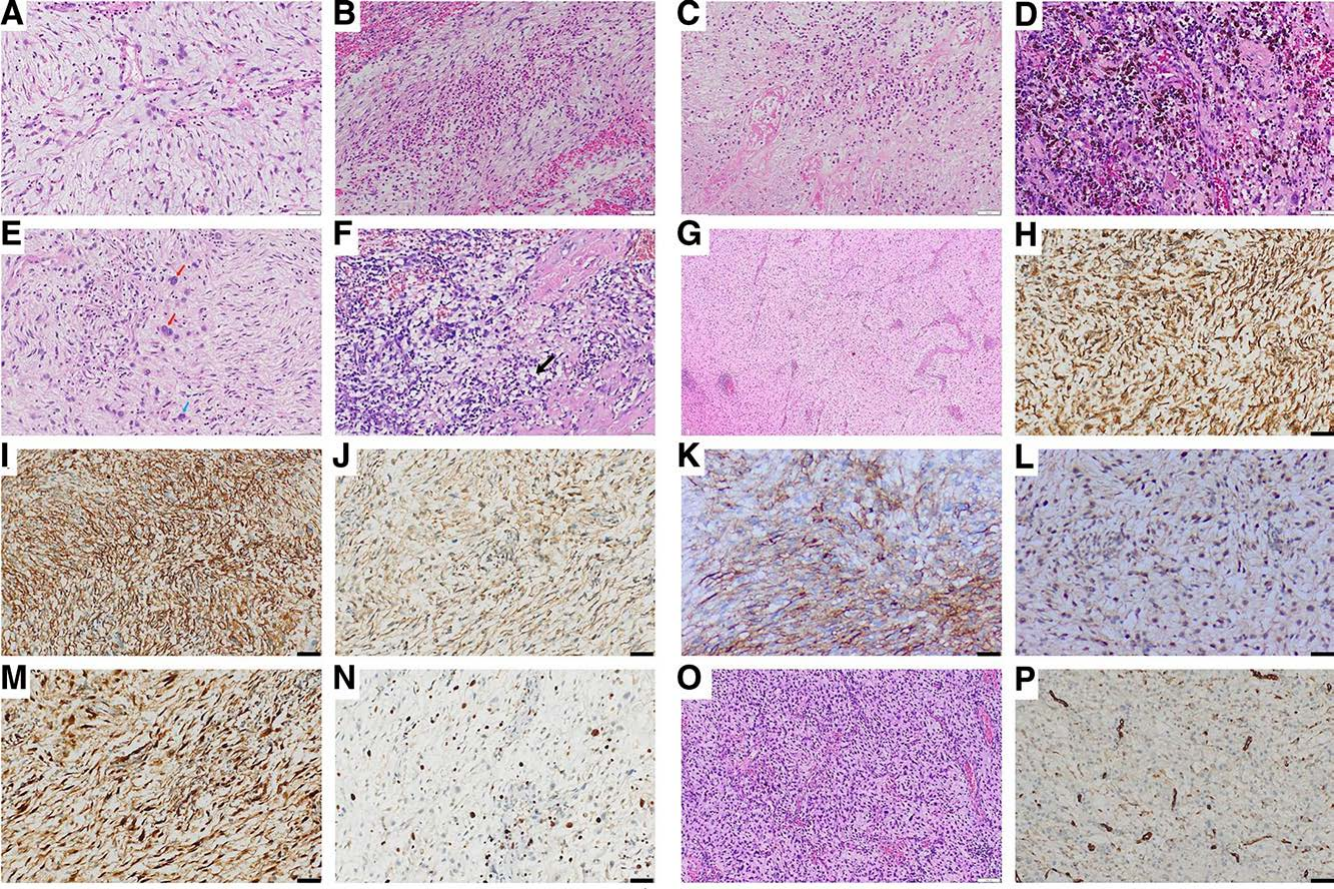
Histopathological features of the tumor. (A) Hyaline areas and myxoid areas (H&E staining, ×200). (B) Inflammatory cells (H&E staining, ×200). (C) Necrosis (H&E staining, ×200). (D) Hemosiderosis (H&E staining, ×200). (E) Multinucleated giant cells (blue arrow) and Reed–Sternberg cells (red arrow; H&E staining, ×200). (F) Pseudo-lipoblasts (black arrow; H&E staining, ×200). (G) Arcuate or curved vascular structure (H&E staining, ×40). (H) Vimentin (IHC staining, ×200; scale bar, 50 µm). (I) CD34 (IHC staining, ×200; scale bar, 50 µm). (J) CD99 (IHC staining, ×200; scale bar, 50 µm). (K) CD10 (IHC staining, ×200; scale bar, 50 µm). (L) cyclinD1 (IHC staining, ×200; scale bar, 50 µm). (M) P16 (IHC staining, ×200; scale bar, 50 µm). (N) Ki-67 (IHC staining, ×200; scale bar, 50 µm). (O) Epithelioid or spindle tumor cells (H&E staining, ×200). (P) CD34 (IHC staining, ×200; scale bar, 50 µm).

A 58-year-old man with no notable medical history presented to our hospital with a 4-day history of persistent pain in the right upper quadrant. During physical examination, no jaundice was found in the patient. The liver and spleen were not affected below the costal margin, and there was no peritoneal irritation. However, the patient had percussion pain in the hepatic area. Laboratory test results revealed increased transaminase levels (ALT, 112.5 U/L; GGT, 128.9 U/L) and a low erythrocyte count (2.84 × 10^12^/L). HBeAb and HBcAb were positive, whereas other commonly used tumor markers, including AFP, CEA, and CA199, were within the normal range. Abdominal computed tomography revealed round, uneven, low-density shadows (11 cm × 11 cm × 9 cm) with clear boundaries in the right posterior lobe of the liver (Fig. 1A). Magnetic resonance imaging (MRI) of the abdomen revealed a low signal on the T1-weighted image and a high signal on the T2-weighted image (Fig. 1B and C). Further, stripy equal signals and necrotic patches were observed. After gadopentetate meglumine injection, the tumor showed progressive enhancement (Figs. 1G and 2). The degree of tumor enhancement was highest during the delayed phase (Fig. 1D) and unequal throughout the hepatobiliary phase (Fig. 1E). Based on these results, we diagnosed it as an intrahepatic malignancy. Thereafter, the patient underwent irregular resection of the liver segments on 6/7/8. The gross specimen showed a lobulated mass measuring 13 × 8 × 7 cm (Fig. 1F), with a gelatinous and fleshy cut surface. After the excision, the lesion was fixed in 10% neutral formalin for 24 hours at 25 ˚C, embedded in paraffin at 56 ˚C, and then cut into 4 µm thick serial sections for hematoxylin/eosin histological staining. Moreover, parallel sections were cut and mounted on silane coated glass for immunohistochemistry, then dewaxed in xylene and rehydrated in graded ethanol. The immunohistochemical procedure was performed using the automated Ventana BenchMark ULTRA platform with primary antibody incubation for 16 minutes at 37 ˚C. The OptiView DAB IHC Detection Kit (Ventana Medical Systems, Inc.) was used to detect protein expression of the primary antibodies shown in Table 1. Finally, all slides were counterstained with Hematoxylin II (Ventana Medical Systems, Inc.) and Bluing Reagent (Ventana Medical Systems, Inc.) for 4 minutes at room temperature. To ensure the reliability of the results of the immunohistochemical reactions, external positive and negative controls were run according to the manufacturer’s instructions. Subsequently, slides were sealed with Permount Mounting Medium and observed under a light microscope (ECLIPSE Ni-U; NIKON CORPORATION). The tumor tissue was composed of a small amount of hyaline areas, a large amount of myxoid areas, and many inflammatory cells, primarily neutrophils (Fig. 3A and B). It showed flaky necrosis and focal hemosiderosis (Fig. 3C and D). Tumor cells were epithelioid or fusiform, with moderately heterotypic nuclei and visible mitotic figures. The lesion also contained multinucleated large cells, Reed–Sternberg cells, and pseudo-lipoblasts (Fig. 3E and F). Furthermore, arcuate or curved vascular structures were observed in the interstitial areas (Fig. 3G). The tumor cells were immunohistochemically stained positively for Vimentin, CD34, CD99, CD10, cyclinD1, and P16 (Fig. 3H–M). The neoplastic cells had a proliferative index of roughly 20% when stained with Ki-67 (Fig. 3N). The tumor cells were negative for CKpan, EMA, S-100, HMB45, CD68, SMA, Desmin, ALK, CD117, DOG-1, Bcl-2, STAT6, LCA, CD30, CD15, and T-cell and B-cell markers (the information for each antibody is shown in Table 1). The patient was finally diagnosed with hepatic myxoinflammatory fibroblastic sarcoma after tumor imaging, microscopic histomorphology, and immunohistochemical analysis. The patient had intermittent radiotherapy and a reexamination within 10 months after surgery, and no abnormalities were found.

**Table 1:** List of antibodies used in our case.

Antibody	Manufacturer	Catalogue number	Dilution
Vimentin CD34 CD99 CD10 cyclinD1 P16 Ki-67 CKpan EMA S-100 HMB45 CD68 SMA Desmin ALK CD117 DOG-1 Bcl-2 STAT6 LCA CD30 CD15 Cell B-cell	ZSGBBIO ZSGBBIO ZSGBBIO ZSGBBIO ZSGBBIO ZSGBBIO ZSGBBIO ZSGBBIO ZSGBBIO ZSGBBIO ZSGBBIO ZSGBBIO ZSGBBIO ZSGBBIO ZSGBBIO ZSGBBIO ZSGBBIO ZSGBBIO ZSGBBIO ZSGBBIO ZSGBBIO ZSGBBIO ZSGBBIO ZSGBBIO	ZM-0260 ZM-0046 ZM-2096 ZA-0526 ZA-0101 ZM-0205 ZM-0166 ZM-0069 ZM-0095 ZM-0224 ZM-0187 ZM-0060 ZM-0003 ZM-0091 ZM-0248 ZA-0523 ZM-0371 ZA-0536 ZA-0647 ZM-0183 ZM-0043 ZM-0037 ZM-0055 ZM-0054	Pre-diluted Pre-diluted Pre-diluted Pre-diluted Pre-diluted Pre-diluted Pre-diluted Pre-diluted Pre-diluted Pre-diluted Pre-diluted Pre-diluted Pre-diluted Pre-diluted Pre-diluted Pre-diluted Pre-diluted Pre-diluted Pre-diluted Pre-diluted Pre-diluted Pre-diluted Pre-diluted Pre-diluted

**Figure 4.**
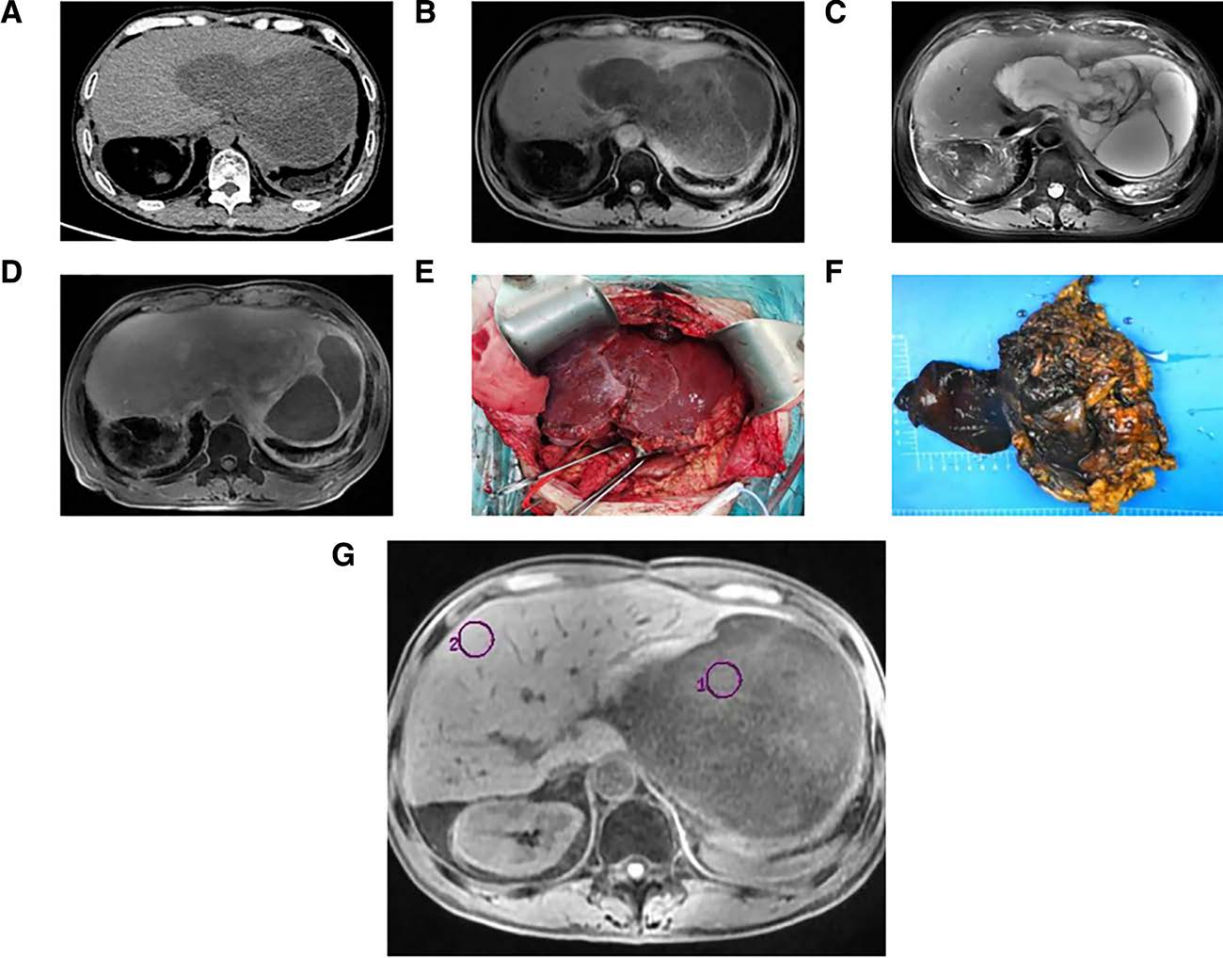
CT, MRI, intraoperative photo and specimen of the tumor. (A) CT image. (B) T1-weighted image. (C) T2-weighted image. (D) Hepatobiliary phase. (E) The intraoperative photo of the tumor. (F) Specimen. (G) The ROI of time–signal intensity curve of the tumor. CT = computed tomography.

**Figure 5.**
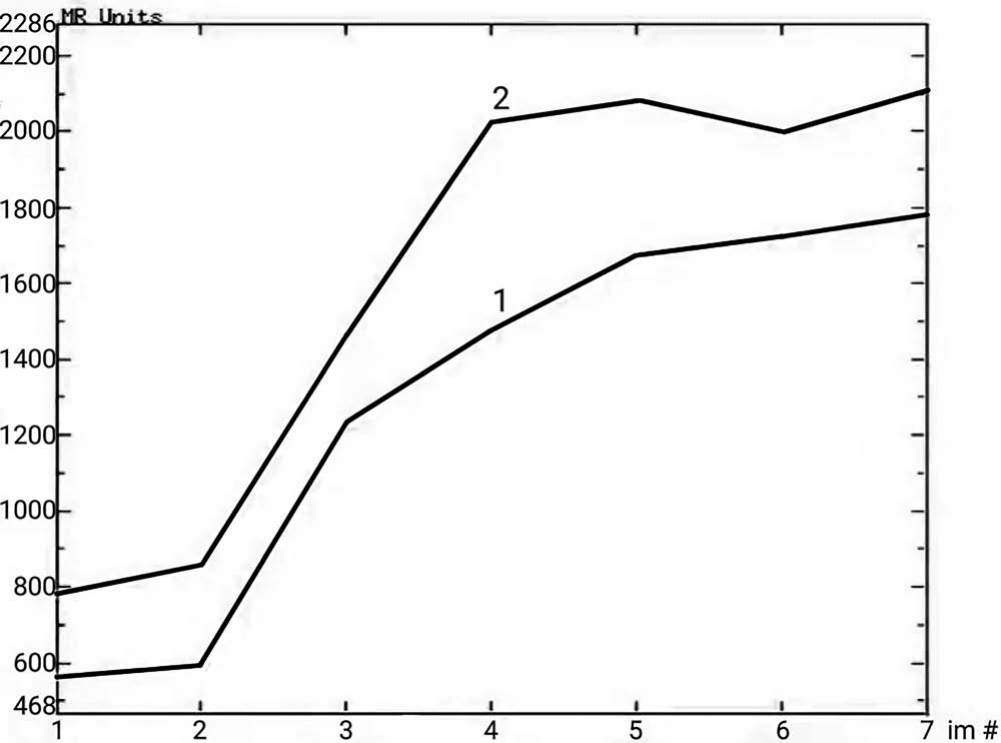
The time–signal intensity curve of the tumor.

However, he returned to our hospital 3 years after the resection complaining of epigastric pain since 3 days. During physical examination, he had tenderness but no rebound pain in the upper abdomen, and no abdominal mass was detected. Laboratory test results indicated increased levels of direct bilirubin (4.8 μmol/L), D-dimer (3.39 mg/L), ferritin (460.100 ng/mL), GGT (116.9 U/L), ALP (129.6 U/L), and white blood cells (9.71 × 10^9^/L) as well as decreased levels of albumin (32.2 g/L) and creatinine (56.2 μmol/L). Computed tomography scan revealed irregular, equal, low and mixed density shadows measuring 18 cm × 11 cm × 14 cm in liver segments 2/3/4. The tumor and gastric wall boundaries were unclear (Fig. 4A). Based on the MRI findings, the lesion was identified as a cystic-solid mass (Fig. 4B and C). After gadopentetate meglumine injection, the cystic component was not enhanced; however, the solid components, tumor wall, and intratumoral septa were significantly enhanced, with a progressive enhancement curve (Figs. 4G and 5). The tumor was considerably enhanced during the hepatobiliary phase (Fig. 4D), similar to the enhancement degree of the hepatic parenchyma, and the local boundary was poorly defined. Based on these results, we considered a postoperative recurrence of hepatic MIFS. The patient once again underwent surgical resection. During the operation, a massive tumor was found on the viscera surface of the liver’s left lateral lobe (Fig. 4E), which was closely adhered to the gastric wall on the side of the minor curvature. The tumor ruptured while separating the minor curvature along the tumor edge, releasing a large amount of jellylike fluid. The excised specimen (Fig. 4F) measured approximately 13 cm × 9 cm × 3 cm in size. The pathological examination of the lesion was carried out in the same way as the first time. The tumor mainly comprises epithelioid or spindle cells arranged in swirls or bundles under the microscope. Moreover, cell density increased, and cellular atypia was more significant than the previous time (Fig. 3O). Immunohistochemical analysis showed diffuse positivity for Vimentin, CD99, CD10, cyclinD1, and P16 and weak positivity for CD34 (Fig. 3P). The Ki-67 index was approximately 30%, whereas other indicators were negative. The patient received multiple pirarubicin-based chemotherapy treatments and an ALK inhibitor (anlotinib) within 6 months after surgery, but the tumor recurred.

## 3. Literature review and analysis

Myxoinflammatory fibroblastic sarcoma is a low-grade malignant soft tissue sarcoma with low metastasis and mortality. Metastasis occurs in about 2% of cases and can spread to regional lymph nodes, lungs, bone, neck, and base of the skull.^[13,14]^ However, the local recurrence rate is relatively high, ranging from 22% to 67%, and recurrence typically occurs 3 to 5 years after the first operation.^[15,16]^ Because the clinical manifestations of MIFS are similar to benign lesions, such as tenosynovitis, ganglion cysts, tenon sheath fibromas, giant cell tumors of the tenon sheath, or fibromatosis, physicians typically do not provide adequate initial treatment, making it easy to relapse after surgery.

At present, many researchers are analyzing the cytogenetic characteristics of MIFS. In 2001, Lambert performed the first cytogenetics analysis of MIFS in the dorsal part of a 53-year-old woman’s foot. The results revealed a t(1;10) (p22;q24) translocation and the loss of chromosomes 3 and 13. Fluorescence in situ hybridization indicated that the gene breakpoint was at the proximal end of Bcl10 and the distal end of GOT1 on chromosome 1p22 and 10q24.^[17]^ In another study on 8 patients with MIFS, Hallor identified 2 genetic pathways in MIFS: t(1;10) resulting in increased FGF8 expression and ring chromosomal amplification of a small segment in proximal 3p associated with VGLL3 overexpression. However, hemosiderotic fibrohistiocytic lipomatous tumor (HFLT) and inflammatory malignant fibrous histiocytoma also showed t(1;10) genetic aberration; in other words, this chromosomal variation is not exclusive to MIFS.^[18]^ In 2011, Antonescu examined 7 cases of MIFS, 14 cases of HFLT, and 3 cases of mixed tumors. The results showed that 83% of cases exhibited simultaneous TGFBR3 and MGEA5 gene rearrangements, indicating that MIFS and HFLT may have a common pathogenesis and the 2 may represent different morphological variations of the same tumor entity or at various stages of tumor growth.^[19]^ Nevertheless, Zreik 2016 study found rearrangement primarily in HFLT and mixed MIFS‐HFLT, with few in pure MIFS, indicating 2 distinct entities and further suggesting that “mixed” tumors may represent a progression of HFLT to a myxoid sarcoma with MIFS features.^[20]^ In addition, Suster evaluated 73 MIFS cases in 2020 and found that some patients with MIFS had BRAF alterations.^[21]^ In 2022, Perret described a novel MIFS variant called “nodular necrotizing” MIFS, including YAP1::MAML2 fusions.^[22]^ Due to differences in results across studies, the final word on the molecular genetic classification of MIFS is not yet available.

MIFS are often lobed and have an irregular texture and color. It is also associated with a broad histological differential diagnosis because the proportion of cellular areas, myxoid lobules, and fibrous inflammatory areas vary for each patient, and MRI results can be nonspecific.^[23–26]^ Immunohistochemically, MIFS cells express vimentin, CD34, CD68, CD10, cyclinD1, calponin, EMA, factor XIIIa, and cytokeratin.^[27]^ Other markers, such as glial fibrillary acidic protein, CD30, CD15, ALK-1, desmin, and leukocyte common antigen, are generally negative.^[28]^ MIFS can exhibit highly variable positive rates for p53, ranging from 0% to 80%, whereas Ki-67 positivity can range from 0% to 20%.^[29]^

The patient could not undergo genetic testing because of financial constraints, and the hospital lacked the necessary facilities for these tests. The clinical manifestations of the patient were nonspecific, with abdominal pain being the primary complaint of the initial and recurring episodes, and both tumor markers were negative. We hypothesized that the patient’s recurrence could be due to several factors: (a) the tumor was closely adhered to the diaphragm before surgery and closely related to the second portal of the liver, making complete removal challenging; (b) the tumor was large; and (c) the patient did not have regular follow-up treatment. Compared with the first time, the recurrent tumor showed an increased proportion of cystic components, a larger volume, and more invasive imaging symptoms. Moreover, the tumor developed significantly to the left, possibly because the stomach is a hollow organ and did not restrict tumor growth. Furthermore, because the tumor was adherent to the stomach wall, it had double blood supply from the stomach and liver vessels, resulting in more rapid growth. Complete surgical resection remains the primary treatment option for MIFS to ensure a negative incisal margin. Furthermore, radiotherapy may help improve local control.^[30]^

This case study presented a sporadic case of MIFS in the liver. The diagnosis of MIFS is based on pathological conformation and a series of laboratory and imaging tests. Preoperative fine needle aspiration cytology may help in MIFS diagnosis.^[31,32]^ The patient mentioned in the study of Shan et al^[33]^ is identical to the one we described. We found no other cases of MIFS in the liver reported in the literature; hence, this patient is the world’s first patient with hepatic MIFS. Compared with the study of Shan et al, we have provided more comprehensive data on imaging, pathological conditions, and clinical treatment of right hepatic lobe tumors and recurrent left hepatic lobe tumors, as well as a more systematic review of relevant literature with greater clinical significance. These findings contribute to a better understanding of the molecular pathological mechanisms of myxinflammatory fibroblastic sarcoma, which will allow for the development of new diagnostic and therapeutic approaches. At this stage, increasing clinical awareness about MIFS is important for clinicians. We should be aware that MIFS can occur not only in the proximal limb, trunk, head, and neck but also affect the abdominal organs.

## Author contributions

**Investigation:** Yize Li, Luyao Zhang, Jing Li, Zhourun Ma.

**Resources:** Xiuchuan Jia.

**Supervision:** Guona Zheng, Xiuchuan Jia, Yingmin Chen.

**Writing – original draft:** Yize Li, Luyao Zhang.

**Writing – review & editing:** Guona Zheng, Xiuchuan Jia, Yingmin Chen.
